# New insights into the treatment of meningoencephalomyelitis of unknown origin since 2009: A review of 671 cases

**DOI:** 10.3389/fvets.2023.1114798

**Published:** 2023-03-15

**Authors:** Nick Jeffery, Nicolas Granger

**Affiliations:** ^1^Department of Small Animal Clinical Sciences, Texas A&M University, College Station, TX, United States; ^2^Bristol Vet Specialists, CVS Referrals & Bristol Translational Health Sciences, University of Bristol, Bristol, United Kingdom

**Keywords:** meningoencephalomyelitis, dog, therapy, glucocorticoid, cytarabine

## Abstract

“Meningoencephalomyelitis of unknown origin” (MUO)—a collective term for a group of clinically-indistinguishable (but pathologically distinct) autoimmune diseases of the CNS—has become increasingly commonly recognized throughout the world. In the 1960s−1980s the focus was primarily on the pathological description of these conditions and, largely anecdotally, their response to glucocorticoids. The subsequent availability of magnetic resonance imaging for companion animals led to a focus on imaging characteristics and response of MUO to various immunosuppressive medications. Previous reviews have not found clear evidence of superiority of any specific treatment regimen. Here, we review outcomes in a further 671 dogs treated with various combinations of glucocorticoids and immunosuppressive drugs and reported since 2009, aiming to determine whether recommendations can be drawn from the material published during more recent decades. We observe that: (i) there is more complete information on outcome of MUO-affected dogs solely receiving glucocorticoids and these reports provide evidence to undermine the dogma that MUO inevitably requires treatment with glucocorticoids *plus* an immunosuppressive drug; (ii) there is far more information on the pharmacokinetics of cytarabine delivered by a variety of routes, revealing that previous dosing and duration of administration in dogs with MUO may not have been optimal; and, (iii) there is a large number of cases that could be available for entry into multi-institutional randomized controlled trials. Finally, we suggest new research avenues that might aid future clinical trials in MUO through improved understanding of etiological triggers and individual patterns of immune response, such as the impact of the gut microbiome, the potential of CSF flow cytometry, and the establishment of robust clinical scores for evaluation of treatment success.

## Introduction

### Terminology of immune-mediated meningoencephalomyelitis in dogs

Immune-mediated meningoencephalomyelitis in dogs has been recognized for at least six decades (1) and what we now label as meningoencephalomyelitis of unknown origin (MUO) was first reported as a distinct disease in dogs in the 1970s (2–4). The term “MUO” is more recent; initial reports refer to the condition as “reticulosis,” which avoided defining it as either neoplastic or inflammatory (4). Subsequently, meningoencephalomyelitis lacking an identifiable etiology was often described as “granulomatous meningoencephalomyelitis” (GME) (5–7) and the first descriptions use this term in preference to the more recent “MUO” umbrella term for the clinical constellation of idiopathic (autoimmune) meningoencephalomyelitis in dogs (8). Although MUO is used as a synonym for autoimmune meningoencephalomyelitis in dogs, it specifically excludes steroid-responsive meningitis [which is recognized as a primary disease of the meninges rather than the central nervous system (CNS) itself] and (the rather uncommon) eosinophilic meningoencephalomyelitis. Conditions such as idiopathic generalized tremor syndrome, idiopathic hypertrophic pachymeningitis or even immune-mediated disease centered on neurotransmitter receptors could conceivably also be part of a spectrum of “MUO” (if defined as “immune-mediated disease of the central nervous system”), but are sufficiently distinct in presentation to be recognized as separate disease entities and are not discussed further here.

During pathological examination it is possible to designate various specific types and severities of meningeal, white and gray matter inflammation and necrosis into subtypes of MUO (8, 9). However, a recent article has implied that pathologic differences may be predominantly of academic interest, because different subtypes can even occur together within the brains of individual affected dogs (10), suggesting there may be little clinical gain from attempting to define the different pathological sub-types in living animals. Instead, in the absence of biopsy confirmation, most cases are treated based on signalment, imaging and cerebrospinal fluid (CSF) findings alone.

### The emergence of meningoencephalomyelitis of unknown origin

Treatment of MUO was limited at first because the cause of the myriad possible neurological signs was unclear and there were reasons for possible confusion with distemper and infectious meningoencephalitis (11). However, during the 1990s MUO was sufficiently well-recognized in the clinic and pathologically described as an idiopathic inflammatory condition that treatment with anti-inflammatory agents was commenced. In one of the earlier summaries of clinical features and outcomes, radiation, which also has a well-recognized anti-inflammatory effect at low doses (12), appeared effective (13). The diagnosis of MUO increased in frequency during the 1990s until the present day, which could be a result of increased recognition, recent increase of popularity of certain breeds (e.g., Pugs, French bulldogs) as well as genuinely increased prevalence. It is difficult to distinguish between these possibilities because this time period also corresponds to that in which veterinary access to magnetic resonance imaging (MRI) rapidly increased. Nevertheless, it is commonly thought that autoimmune disease is likely becoming more prevalent in pet dogs in the West, as it is in people (14); and, in dogs, may also be caused through poorly controlled breeding that reduces genetic diversity (15). It is therefore probable that this is genuinely a more common disease than it was in the 1980s and before.

### Parallel disease in humans

Non-infectious (immune-mediated) meningoencephalomyelitis is common in humans [e.g., neurosarcoidosis, neuromyelitis optica, acute demyelinating encephalomyelitis, etc. (16)] and most widely apparent in the high prevalence (especially in specific geographical regions) of multiple sclerosis (MS) (17–19). The clinical signs of MS are similar to those of MUO in dogs, so that many different syndromes are recognized to be part of a constellation of clinical signs. In MS the most common presentations relate to visual deficits and spinal cord dysfunction [which also occur, albeit at lower frequency, in MUO-affected dogs (20)], but cognitive deficits have become more widely appreciated recently (17, 21). Pathologically, MS in humans and MUO in dogs also are broadly similar, consisting of inflammatory cell infiltration, often along vascular pathways; in MS both T and B cell infiltration is recognized, whereas MUO appears to be predominantly T cell dominated (22). The recognition of association of dog leucocyte antigen class II and necrotizing meningoencephalitis (in pugs) also demonstrates strong parallels with aggressively progressive forms of MS (23). On the other hand, demyelination—the hallmark pathological feature of MS—is rarely a dominant feature in MUO (8), although the recognition of regions of Schwann cell remyelination in MUO lesions (24), provides clear evidence that it does, at least sometimes, occur.

Because of the huge importance of MS in human neurology there are laboratory models of the disease in experimental animals, collectively known as “experimental autoimmune encephalomyelitis” (EAE) (25). This is a rapidly-developing paralytic disease of rats and mice (and can also be created in other species) that is induced by injection of processed components of the CNS together with an adjuvant to trigger widespread inflammation in the CNS. Pathologically, this condition bears even closer resemblance to MUO in dogs than MS in humans, for instance in the lower prominence of demyelination. The parallels in terms of neurological signs and pathological features of both EAE and MS with MUO can be useful in determining useful therapeutic strategies for affected dogs.

### Contributing factors and etiology of MUO

The etiology of MUO is by definition unknown but, in addition to individual genetic predisposition, many autoimmune diseases are strongly suspected of having a trigger, which may be infectious, environmental or neoplastic (26). In contrast to recent developments in MS (27, 28) and despite extensive searches for infectious triggers, none have so far been identified for MUO in dogs (29–36) and neither have broad environmental risks been identified in association with canine immune-mediated disease more generally (37), and so the etiology is assumed to be an idiopathic self-directed immune response. Nevertheless, the complicated immune-mimicry that underpins the relationship between Epstein-Barr virus and MS in humans implies that a similar relationship between an as yet unidentified pathogen and MUO cannot be ruled out. Detailed case-control studies on large populations of dogs would be required to identify such relationships.

Genetic causes have long been suspected in dogs with MUO because of the high prevalence in specific breeds, namely Pug, Maltese, Yorkshire terrier, Pekingese, Chihuahua, Papillon, Shih tzu and others (38), and the underlying genetic risk factors in these breeds are beginning to become more apparent: (i) ~75% of Pugs with necrotizing encephalitis have a specific combination of alleles coding for major histocompatibility complex class II molecules on chromosome 12 containing exons for the dog leukocyte antigen and conferring on them a relative risk of 5.45 of developing the disease (39); and (ii) in Maltese dogs, *ILR7* on chromosome 4 and *FBXW7* on chromosome 15, both involved in the immune system regulation, could be implicated in necrotizing encephalitis (40).

## Aim of the review

There remains considerable controversy around treatment of MUO in dogs, because a multitude of drugs have been prescribed, using many different dosing schedules and combinations. In this review we summarize the data available from publications since 2009 to follow-up on our previous review (38). The objectives of this current review are to assess the treatment results, in particular for those cases receiving glucocorticoids alone, and those reported in association with various dosing schedules of cytarabine, and how these correspond to recent evidence from pharmacological investigations. In addition, we suggest new research avenues that could investigate the etiology of the condition and may guide future treatment.

## History of treatment of MUO

Because no specific directly-treatable etiology has been identified, treatment almost exclusively focuses on immunosuppression, although there is frequently a need for adjunctive treatment of associated neurological signs, such as seizures [that occur in 20–25% of cases (41–43)]. In this review we will focus only on treatment aimed at the underlying disease process rather than symptomatic therapy aimed specifically at controlling the resulting clinical signs.

When non-infectious meningoencephalomyelitis was first diagnosed, affected dogs were treated with glucocorticoids (GCs), predominantly dexamethasone, but often for relatively short periods of time and, upon relapse, were frequently euthanased—thereby permitting the diagnosis (11, 13, 38). In a large early study, some cases were also treated with radiation, which the authors associated with more prolonged survival after diagnosis (13). With hindsight, this was an unusual case series because the median survival period for dogs with multifocal disease (a common presentation) was extremely short (14 days, ranging from 1 to >1,215 days), which would nowadays be considered unusual, although it may also be at least partly attributable to the study inclusion requirement for definitive *post-mortem* diagnosis. Nevertheless, as stated by Zarfoss et al. (44), the prognosis at that time was widely considered to be poor, with Thomas and Eger (45) stating that most dogs with GME were euthanased or died within 3–6 months of presentation.

Glucocorticoids have been the mainstay of treatment in the intervening years, although the positive response to immunosuppression combined with the prominent adverse effects of high-dose GC therapy have prompted searches for substitutes. A large range of immunosuppressive therapies have been used in treatment of MUO (38, 46) with little apparent overview of what attributes of the medications might be desirable; for instance, which specific aspects of the immune response should be targeted. Cytosine arabinose (i.e., cytarabine) has become a favored medication for MUO, since its first use by Nuhsbaum et al. (47) and presentation by Cuddon at the 20th ACVIM forum in 2002 (48), for reasons that appear opaque. As with many other conditions in companion animals, there is a dearth of evidence on comparative efficacy of different drug regimens, because formally-designed randomized comparative trials are rare. Instead, veterinarians have tended to take the alternative approach of using a specific medication with which they feel comfortable and then, if it fails, to introduce another medication. This can be satisfactory as a way of managing individual dogs but is unsatisfactory in designating specific therapies as likely to be the optimal starting medication after diagnosis.

## Updates of outcomes associated with specific medications

In our previous review (38) we noted that several immunosuppressive medications had been used for treatment of MUO, with little evidence to support any specific regimen over another. Since that time, more data has become available on responses to specific medications and specific regimes, although no formal trials of one medication vs. another have been conducted. Almost all protocols continue to use GCs as the first-line treatment, followed by addition of various other medications in attempts to spare GC use or to provide additional control of clinical signs, or both. We have searched the literature from 2009 onwards, using the same methods and key words described in our previous review [see (38)] and identified 15 studies for further review (Table 1). Here we summarize the results of treatment of any form of MUO as included by the authors. One publication detailed the outcomes for a contingent of dogs that presented with spinal cord signs only (62) and included dogs treated with GCs and/or cytarabine, with a median survival of 669 days. This report is not discussed below because of the clear distinction in presentation, although there is currently little reason to suppose that outcomes differ with different distributions of lesions at presentation.

**Table 1:** Summary of treatments and outcomes in dogs with meningoencephalitis of unknown origin published since 2009.

**Medication(s)**	**References**	**Combined with other drugs**	**Dose and delivery method**	**Study design**	**Number of dogs**	**Comment**	**Survival data: median and range in days (if not stated differently)**	**Previous survival findings prior to 2009 [see Granger et al., (38)], median and range in days**	**Number of dogs previously reported for each medication**
Methylprednisolone and prednisolone	Mercier et al. (49)	No	IV methylprednisolone 30 mg/kg then 15 mg/kg 3 h later, then 10 mg/kg 2 h after that, followed by prednisone at 2 mg/kg per day	Prospective case series (3 dogs classed as non-responders received adjunctive therapy)	16	3 dogs classed as non-responders received adjunctive therapy	602 (45–654)	36 (2–1,200)	26
Dexamethasone and prednisone	Paušová et a. (42)	No	IV dexamethasone 2 mg/kg per day for 3–5 days, then prednisone at decreasing dose starting at 2 mg/kg per day	Retrospective cohort study	168	14 dogs euthanase before treatment; relapse occurred in 19 dogs during therapy and in 45 after stopping therapy	570 (2–3,540)		
Dexamethasone and prednisone	Lawn and Harcourt-Brown (50)	No	Per os prednisolone 2 mg/kg per day (or IV dexamethasone equivalent) for at least 3 weeks and then tappered down over at least 3 months	Non-randomized retrospective cohort study	63	7 dogs died in the first 7 days and 44 were alived at 100 days, which was the time frame analyzed	44 of 63 dogs (~70%) alive at 100		
Mycophenolate	Barnoon et al. (51)	Glucocorticosteroids	20 mg/kg twice a day	Retrospective cohort study	25	5 dogs developed gastroenteric signs	250 (6 to >1,679)	118 (10–240)	4
Mycophenolate	Woolcock et al. (52)	Glucorticoides, cytarabine and cyclosporine	20 mg/kg once a day	Retrospective cohort study	25	2 dogs had mild adverse affects	731 (43–1,672)		
Mycophenolate	Song et al. (53)	Glucocorticosteroids	20 mg/kg twice a day	Retrospective cohort study	86	50% of dogs had adverse effect mainly gastroenteric signs	558 (3–2,634)		
Cytarabine	Lowrie et al. (54)	Glucocorticosteroids	50 mg/m^2^ twice a day at progressively longer intervals starting from 3 weeks	Prospective case series (13 dogs died of were euthanased within 3 days)	39	13 dogs died of were euthanased witing 3 days	26 (0–2,250)	Median survival of 384, 519 and 531 days in three studies, ranging from 46 to 1025 days	30
Cytarabine	Lowrie et al. (55)	Glucocorticosteroids	First cytarabine treatment of 100 mg/m^2^ over 24 h then same protocol as Lowrie et al. (54)	Prospective case series (4 dogs died or were euthanased within 3 months)	41 [compared to the 39 dogs from Lowrie et al. (54)]	4 dogs died of were euthanased by 3 months	37 of 41 dogs (~90%) alive at 12 months		
Cytarabine	Stee et al. (56)	Glucocorticosteroids	Single 100 mg/m^2^ cytaratine injection over 24 h	Prospective case series	42 [compared to the 41 dogs from Lowrie et al. (55)]	6 dogs died of were euthanased over 36 months	28 of 42 dogs (~67%) alive at 34 months		
Cytarabine	Barber and Downey Koos, (57)	Glucocorticosteroids and cycosporine	Single 200 mg/m^2^ cytaratine injection or given in 4 doses subcutaneously over 24 h	Retrospective cohort study	21	5 dogs died or euthanased, median survival was 555 days	16 of 21 dogs (~76%) alive at 36 months		
Cytarabine	Lawn and Harcourt-Brown (50)	Glucocorticosteroids	200 mg/m^2^ cytaratine injection over 8–12 h every 3–4 weeks	Retrospective cohort study	27	10 dogs died or euthanased within 100 days, which was the time frame analyzed	17 of 27 dogs (~63%) alive a 100 days		
Lomustine	Flegel et al. (58)	Glucocorticosteroids	~60 mg/m^2^ every 6 weeks	Retrospective cohort study	24	Possible anemia; leucopenia in one dog	457 (107–709) in 14 dogs with granulomatous meningoencephalomyelitis and 329 (98–628) in 10 dogs with necrotising encephalitis	Median survival of 287 and 335 days in two studies, ranging from 150 to 740 days	15
Ciclosporin	Pakozdy et al. (59)	Glucocorticosteroids	3 mg/kg twice a day	Non-randomized retrospective cohort study	14	Gastrointestinal hemorrhage requiring transfusion in one dog	620 (8–870)	Median survival of 240, 423 and 930 days in three studies, ranging from 6 to 1,290 days	23
Ciclosporin	Brady et al. (60)	Glucocorticosteroids; 4 dogs also had cytarabine	3–5 mg/kg twice a day	Retrospective cohort study	40	Gastrointestinal signs	1,345 (487–?)		
Azathioprine	Wong et al. (61)	Glucocorticosteroids	2 mg/kg once a day	Retrospective cohort study	40	No side effect related to medication	1,834 (50–2,469)	Not previously reported	0

A point of comparison is provided in the far right last two columns: in these are reported the median (and range) survival obtained from our previous review and extracted from publications available before 2009 to provide comparison with survival times reported since 2009 in the new papers reviewed. The ? means unknown.

### Traditional therapeutic approaches

#### Glucocorticoids as sole therapy

Although there is considerable weight of opinion that sole use of GCs is not as efficient a therapy for MUO as more complicated regimens there is, at present, no definitive evidence in support of such claims. Furthermore, it is often difficult to distinguish relative effects of other agents because they are almost exclusively used together with GCs and, in many instances, are not commenced until after a specified period (during which many severely affected dogs may already have succumbed to the disease or improved so as not to be treated with a second medication). In our previous review (38), we found only 26 dogs treated solely with GCs and these had a reported median survival of 36 days, possibly explaining the belief that treatment with GCs alone is inappropriate. However, the survival of those dogs was extremely variable, ranging from 2 to 1,200 days.

Since 2009, three groups have provided much additional data on sole therapy of MUO with GCs. Mercier et al. (49) reported on 16 dogs that received intravenous methylprednisolone at 30 mg/kg then 15 mg/kg 3 ho later, then 10 mg/kg 2 h after that, followed by prednisone or methylprednisolone at 2 mg/kg/day. Seven were designated as “responders” based on second CSF analysis and three non-responders received adjunctive therapy. They recorded a median survival of 602 days for all included dogs. Paušová et al. (42) reported on a cohort of 168 dogs, although it is important to note that 14 dogs that could have been included were euthanased upon diagnosis (therefore implying that subsequent survival analysis is biased by their exclusion) and these received intravenous dexamethasone at 2 mg/kg/day for 3–5 days, then prednisone at decreasing dose starting at 2 mg/kg/day. Relapse occurred in 19 dogs *during* therapy and in 45 *after* stopping therapy. Median survival time was 570 days (the dogs that were immediately euthanased upon diagnosis were not included in this analysis). Lawn and Harcourt-Brown (50) also reported short-term outcomes on 63 dogs that had received GCs alone in a non-randomized retrospective cohort study. Following an initial dose of 2 mg/kg/day prednisolone (or dexamethasone equivalent) seven of 63 dogs (11%) died within 7 days, which was equivalent to the proportion dying in a cytarabine and GCs group at the same institution (see below), and 40 dogs were alive at the 100-day study termination point.

#### Mycophenolate

This immunosuppressive medication had been reported in only 4 dogs for treatment of MUO in our previous review and there are now three further reports.

Barnoon et al. (51) reported on 25 dogs that were given 4 mg/kg/day prednisone as soon as possible after diagnosis, plus 20 mg/kg mycophenolate twice daily; doses were tapered with time. Five dogs showed complications thought to be caused by mycophenolate, which was then withdrawn and these dogs were not included in survival outcomes. For the remaining 20 dogs, median survival was 250 days. Apart from acute problems with gastroenteric signs the long-term adverse effects were not severe, although there were five recorded instances of disease that may have been induced by long-term immunosuppression.

Woolcock et al. (52) reported on 25 dogs (although a further two were excluded through euthanasia because of cost / prognosis at <7 days). Each dog received prednisone at 2 mg/kg/day or more, plus mycophenolate at 20 mg/kg per day. Some dogs also received cytarabine and / or cyclosporine and other medications. Median survival was 731 days. Two dogs showed mild adverse effects thought to be associated with mycophenolate.

A publication by Song et al. (53) included 86 dogs for follow-up (although eight were removed because of adverse effects of mycophenolate); each was given prednisone at 2 mg/kg/day plus mycophenolate at ~20 mg/kg twice daily. Median survival for all dogs was 558 days. Almost 50% dogs had adverse effects from treatment—mainly gastrointestinal signs acutely but then long-term evidence of adverse immunosuppressive effects (infections) and hematological abnormalities (~50% dogs).

#### Cytosine arabinose

Cytosine arabinose (i.e., cytarabine) has been used as a therapy for MUO first in 2002 in one dog (47) and started subsequently to be reported in case series from 2006 (44) and 2008 (63). Many of the subsequent clinical investigations of efficacy reviewed here have originated from the group lead by Lowrie in the UK and which included dogs free from other medications before diagnosis of MUO. The first of this series of publications (54) reported on 39 dogs in which treatment, similarly to Zarfoss et al. (44) and Menaut et al. (63), consisted of a combination of prednisolone commencing at 2 mg/kg/day for 4 weeks and decreasing thereafter, plus cytarabine at 50 mg/m^2^ twice daily subcutaneously for each of 2 days at progressively longer intervals (i.e., 3 weeks, then 4 weeks, 5 weeks etc.). Thirteen dogs died or were euthanased within 3 days of diagnosis and the overall median survival was 26 days. A follow-up study by the same group (55) compared the outcomes reported in 2013 with outcomes of a similar second cohort of 41 dogs that received the same GC regimen but initially received cytarabine as an intravenous dose of 100 mg/m^2^ over 24 h rather than subcutaneously (but subsequent treatments were the same). The outcomes were strikingly different, in that only four dogs of the new cohort died or were euthanased by 3 months (compared with 22 in the subcutaneous cytarabine cohort) and all the remaining 37 dogs were still alive at 12 months. In the third report in this series (56) a cohort of 42 dogs was treated with the same GC regimen plus a single cytarabine 24-h continuous rate infusion (CRI) of 100 mg/m^2^ at diagnosis. Treatment response appeared good, with 28/42 dogs reported as successful at 34 weeks and, overall, six dogs died (or were euthanased) because of their disease during a follow-up of 36 months. Median survival time was not available for each of these last two reports because so large a proportion of dogs survived, but the median time to relapse was similar (299 vs. 285 days, respectively) between those that received only a single cytarabine dose and those that received that plus subsequent subcutaneous doses.

This interesting series of reports raises many questions. First, “historic controls” are controversial, the problem being that it can be difficult to ensure that the populations recruited at different time periods are truly similar at entry to the study and that subsequent clinical decisions are made in the same way in each study sample. Broadly, in this series the study populations can be seen to be similar (although “how similar?” cannot be properly evaluated by statistical testing), but this cannot exclude the possibility of “unmeasured confounders” that, in contrast, will also be equivalent in sample populations in large randomized trials (the gold standard for such comparative studies). The median survival in the first report was unusually short for a modern cohort of MUO cases; such unusual results are more common when small populations are analyzed and are difficult to interpret without a randomly-allocated contemporary control group.

Second, a reasonable conclusion from this series of studies would be that cytarabine has little overall benefit beyond that given by GCs because the repeated dosing in the first two studies did not have benefit over the single dose given in the third study [this conclusion is also supported in the way cytarabine is used in humans, in whom it is administered over many consecutive days]. The possibility remains from this series of reports in dogs that the single initial dose has benefit, but recent data has called that too into question. In a non-randomized retrospective study in which dogs received prednisone and cyclosporine at the time of diagnosis either with or without cytarabine (200 mg/m^2^ as a CRI, or as four subcutaneous injections over 2 days) there was no apparent benefit of the cytarabine (57). Similarly, Lawn and Harcourt-Brown (50) did not detect benefit of cytarabine in their retrospective cohort study. However, it must be considered that the possible benefit of cytarabine in those reports might have been masked by the (beneficial) effect of concurrent GCs and/or cyclosporine but this finding implies there is little or no additional benefit.

Lastly, there is also the question of whether the administered dose of cytarabine was sufficiently high to achieve therapeutically useful blood levels. This specific issue has been examined in many recent publications but, specifically, it was shown that 200 mg/m^2^ [double the dose used in (55)] CRI cytarabine over 24 h was unreliable at producing durable blood levels above 1μg/mL (the presumed therapeutic level) (64). Nevertheless, whether the efficacy of cytarabine is time- (above threshold) or concentration-dependent is still not established. A reasonable overall conclusion is that the small case numbers included in many of the reports implies greater likelihood of more extreme results and so the reported differences between outcomes in different study protocols are likely to represent random variation rather than true differences in effects of different treatment regimens.

##### Pharmacokinetic studies

Important recent work has focused on cytarabine pharmacokinetics, especially that related to administration by different routes. This is especially pertinent because intravenous infusions imply a high cost to the owners (because it is typically given over at least 8 h and requiring hospitalization of the animal), thereby incurring an elevated risk of financially-driven euthanasia of affected animals. Many questions remain regarding the optimal method of delivering cytarabine for treating dogs affected by MUO. Many of these are directed at the important question of whether it is possible to use this therapy without needing the owners to attend with their dogs in person for intravenous injections.

The use of cyatarabine is predicated on the notion that it is necessary to attain a blood level of 1μg/mL (64), based on its uptake by cancer cells *in vitro* (65) resulting in their death (66). However, data from human medicine suggest that CSF:plasma ratio of cytarabine is ~10–25% and that intravenous infusions of 400 mg/m^2^ are required to attain a CSF concentration of 1.2 mmol (approximately that required for cytotoxicity) (67). Most veterinary studies on cytarabine regimens have been conducted to determine how well they attain concentrations of 1μg/mL, although it remains unknown how long it is necessary for the blood level to exceed that value.

Fortunately, the consensus from these studies is that (modeled) repeated subcutaneous injections can attain just as prolonged periods of high blood concentrations as intravenous delivery (68, 69) and a single high-dose subcutaneous injection provides a (marginally) higher overall drug exposure (in blood) than repeated lower dose injections (70). A recent technological development (the “Omnipod” system) opens the possibility of delivering high dose cytarabine *via* subcutaneous infusions that can be monitored by owners at home (71).

#### Lomustine

Flegel et al. (58) reported on 43 dogs with various categories of MUO that received prednisone at variable dosage (some quite high), plus lomustine at a median dose of ~60 mg/m^2^ every 6 weeks. Median overall survival is difficult to extract from this report because the survival was categorized by the individual sub-type of MUO, but all were between 91 and 457 days. Adverse effects appeared to be limited, although there was a tendency for treated dogs to show anemia; one dog developed leucopenia and died of septic shock (this complication was attributed to lomustine).

#### Ciclosporin

Pakozdy et al. (59) reported on 14 dogs, some treated with ciclosporin 3 mg/kg twice daily as well as GCs (which was often given at high dose). Median survival with ciclosporin was 620 days (vs. 28 days for GCs alone). Adverse effect in one ciclosporin dog was severe: life-threatening gastrointestinal hemorrhage that required a blood transfusion. The comparison with GC treatment alone is difficult to interpret because the two regimens were not parallel or allocated randomly.

Brady et al. (60) included 40 dogs that were treated with prednisone at 1 mg/kg/day and often started with intravenous dexamethasone; ciclosporin (3–5 mg/kg twice daily) was added to the treatment regimen and the prednisone dosage increased to 2 mg/kg/day when the infectious disease results were known, although no dogs were lost to treatment or survival analysis through this mechanism (Brady, personal communication). Median survival was reported to be 1,345 days; few dogs died in the first few weeks after diagnosis (in contrast with most other studies) and, notably, the shortest survival was 487 days. Four dogs had cytarabine added (one because of ciclosporin adverse effects). No other adverse effects were reported.

#### Azathioprine

Wong et al. (61) reported on 40 dogs treated with prednisone 2 mg/kg/day plus azathioprine at 2 mg/kg/day, followed by recommended decreasing dosage, and some dogs also received dexamethasone. Azathioprine was only commenced after diagnostic tests were complete. Median survival of the included dogs was 1,834 days and dogs that died soon after diagnosis and before initiation of azathioprine were not included in this analysis. Few adverse effects that could be attributed to azathioprine were recorded, and even those that might have been attributed to azathioprine were generally mild.

### Non-traditional therapeutic approaches

#### Stem cells

Stem cells have long been noted to show anti-inflammatory effects and have previously been used for this effect in experimental models and human autoimmune disease [e.g., (72)]. In 2015, Zeira et al. (73) reported on a series of eight dogs that received autologous bone marrow mesenchymal stem cells after failing to respond adequately, or having developed adverse effects owing, to conventional therapy with GCs, with or without adjunctive cytarabine. The cells were administered intrathecally and *via* systemic blood vessels (carotid artery or intravenous). The dogs were reported to gradually improve over a 6-month period and the seven long-term survivors (one dog died of intercurrent disease) became neurologically normal or near-normal, although were also continuing to receive prednisone.

#### Radiation

Low-dose radiation has an anti-inflammatory effect and there are a few reports on its use for MUO. Recently, Beckmann et al. (74) delivered 30 Gray over 10 fractions in 2 weeks to the whole brain or the lesioned region of six dogs that completed the therapy (one other was withdrawn because it probably had another diagnosis). Five dogs improved and one was unchanged at the end of therapy. Radiotherapy was initiated at between 3 and 56 days after starting medical therapy with prednisone at ~2–4 mg/kg/day and, in one case, cytarabine. Twelve-month outcomes were good in five of the six cases (one relapsed and was euthanased), with normal or near-normal neurologic status.

#### Intrathecal cytarabine

Although cytarabine readily crosses the blood-brain barrier there can be advantages to intrathecal (i.e., subarachnoid space) delivery because it allows higher concentrations to be available to cross the CSF-brain barrier and longer half-life. Subarachnoid delivery of cytarabine is used in human medicine for treating various malignancies and has a good safety record (67). Genoni et al. (75) used intrathecal delivery in a series of 112 dogs with MUO at 100 mg per dog (a calculated dose range of between 70 and 450 mg/m^2^), and in some animals this was accompanied by methotrexate (2.5 mg per dog). One dog developed seizures that were easily controlled. Whether the treatment was of benefit is not recorded and it is not reported whether the intrathecal treatment was repeated. According to a study of cytarabine administration to rat brain (76), intrathecal delivery would appear to have little benefit over systemic administration in achieving widespread high brain tissue concentration. Although intrathecal delivery does circumvent the blood-brain barrier it still does not place drug directly into the brain and traverse of the CSF-brain barrier is still required. Instead, the advantage may lie in the ability to achieve high CSF concentration for a prolonged period (because liver degradation is avoided) but it appears that depth of penetration into brain tissue is still limited.

## Discussion

### Where are we now with treatment of MUO?

The conclusion of our previous analysis of treatment for MUO was that there was no specific regimen that appeared to show overall superiority compared with others. It appears that, despite the addition of more recent reports, we are still in much the same position now. There are many protocols that have been used by different centers, but no direct comparisons between different approaches.

Glucocorticoids as a single therapy have now been reported in a larger number of dogs (26 cases before 2009 vs. 247 cases since 2009) compared to our previous review and the filling of this knowledge gap suggests survival periods of similar magnitude to dogs receiving GCs *plus* an adjunctive therapy and with extremely wide ranges, although direct comparison between studies is difficult. Despite the use of a wide range of different immunosuppressive agents there is still not reliable evidence that any of these are superior to GCs alone and there are potential benefits to owners to relying upon GCs alone, such as the ease of access, cheap cost and ease of administration. Nevertheless, this must also be balanced against the high rate of adverse effects of high-dose GC administration. Even so, there is also the potential for many owners to request euthanasia of their affected dog because of the costs associated with frequent revisits (for cytarabine injections or for blood tests). Financial exhaustion is a common cause of euthanasia in veterinary medicine (77).

Radiotherapy appears likely to be effective based on the small numbers of cases reported, in that it appears able to “rescue” dogs that had previously been progressing poorly, but might not be accessible—geographically or financially—for many owners. Furthermore, it can be difficult to commence radiotherapy as an emergency treatment, at least in some geographical locations, but is often required because of the recognized high rate of death soon after diagnosis (38, 46). Dogs in the recent report (74) had been treated medically for between 2 and 35 days before starting radiotherapy which suggests the possibility that the included cases were (inadvertently) selected for longevity (because of the need to survive for at least 48 h after diagnosis). Nevertheless, this same caveat also applies to many reports on medical therapies.

It would appear that the reliance on cytarabine perhaps mainly derives from a small number of cases reported in the mid-2000s, in which a subcutaneous dose of 50 mg/m^2^ twice a day for 2 days and then repeated every 3 weeks was empirically administered, perhaps because it appeared against a background of an expected largely dismal prognosis. Although specific evidence for efficacy is lacking, there is evidence that it is not associated with a high incidence of adverse effects (78), possibly reflecting the low dosages used in MUO cases. Subsequent recording of prolonged survival of MUO-affected dogs when treated with many other immunosuppressive regimens (including GCs alone) imply that the cytarabine regimen used in these reports may not have been as efficacious as originally interpreted. For instance, treatment also included concurrent prednisone (1–2 mg/kg twice daily) as well as commencing with a high dose of dexamethasone. Moreover, more recent publications (see above) examining the pharmacokinetics of cytarabine raise questions about the appropriateness of previous apparently-effective dosing regimens, including that reported by Zarfoss et al. (44), implying that the impact of this therapy might be difficult to extract from the overall immunosuppressive effect of the high GC dose. Of course, it may be that cytarabine is specifically useful but further evidence is needed regarding the ideal dose and dosing interval. An argument against cytarabine as an agent in MUO is that there are many protocols that have been advocated but there is little that is known about how best to dose this medication, and specifically whether cytarabine crosses the blood-brain barrier *via* time- or concentration-dependent mechanisms. Cytarabine is primarily administered in people (typically for treatment of acute myeloid leukemia) *via* twice daily subcutaneous injection or daily intravenous infusion for at least 7 consecutive days (79, 80) using doses ranging from 100 to 200 mg/m^2^, creating prolonged high blood concentrations (67) and this could perhaps be explored further in MUO. There appears to be plenty of scope to increase the dose because, so far, few adverse effects have been reported (78). Rescue protocols in people consist of administration of enormous doses up to 3,000 mg/m^2^, with 1,000 mg/m^2^ being seen as a safer approach (81). Many of the veterinary regimens seem to have marginal probability of reaching the therapeutic threshold required to achieve adequate CSF concentration. On the other hand, in human medicine there is evidence that the therapeutic ceiling (for acute myeloid leukemia, at least) is reached with quite low doses (200 mg/m^2^) anyway and so higher doses might just expose patients to higher risk of adverse events for no benefit (81).

Adverse effects of medications do not seem to be limiting for most of the immunosuppressive agents used, with the possible exception of high-dose mycophenolate (i.e., when used at 20 mg/kg twice daily), which appears to be associated with a relatively high rate of unacceptable complications. Glucocorticoids themselves are probably responsible for most of the adverse effects we see on a day-to-day basis. Indeed, the rationale for using most of the other immunosuppressive agents is largely to avoid the long-term effects of the GCs, which do themselves often “work” very well in this condition.

In comparison with therapy for MS in humans (see below) the only medication apart from high-dose GC therapy that is available and similar in veterinary medicine is leflunomide. In MS, teriflunomide (the active metabolite of leflunomide) is the only agent to which veterinarians have routine access that is considered to have value in treatment of MS (82). There are, currently, few reports on use of leflunomide in MUO (83).

### Where might we be going with MUO treatment?

#### Comparisons with MS in humans

As mentioned above, there are many reasons to consider MUO as “dog multiple sclerosis” although the analogy is imperfect because, as mentioned above, there are differences in pathological detail between dogs and humans. However, there are even differences in character and severity of EAE between different strains of rat, attributable to differences in genetic or environmental background, or both (84–87). Therefore, it is perhaps not unreasonable to consider that differences in lesion detail resulting from a parallel immune phenomenon in the two species may result from the large differences in genetic and environmental factors between humans and dogs. While MUO may have some value as a model condition for human medicine, conversely, because of the considerably more advanced knowledge of pathogenesis of MS and the recent rapid development of new therapies for affected humans, it is more realistic for veterinarians to consider human MS as the model disease for MUO. Several different subtypes of MS are recognized, based on their progression dynamics and, following this approach, we might consider that MUO is most similar to primary progressive MS, which has a relatively poor prognosis in humans (17).

In primary progressive MS, although GCs may be used initially, the mainstay of therapy has now been revolutionized by monoclonal antibodies that modulate immune cell function. Following successful clinical trials, ocrelizumab, a monoclonal antibody against the CD20 antigen on B cells, is now the recommended first-line treatment (82, 88). Other, more veterinary-accessible, agents have either been shown not to be useful [glatiramer acetate (89)] or have uncertain benefits [interferon-beta (90, 91)] in clinical trials in human primary progressive MS.

The problem for veterinarians is that monoclonal antibodies could be applied in dogs [as they have been for dermatologic disorders (92)], but there are many obstacles of time, cost and the relatively small market for these products, that might limit our access in future to parallel “canine-ized” medications for treatment of autoimmune CNS disorders.

#### Fecal microbial transfer

In 2011, following a tradition of modeling MS with the experimental disease EAE in rodents (25), it was found that germ-free rats are far less susceptible to development of EAE than those that have a normal gut microbiome (93) and subsequent exploration has revealed that there are gut bacteria that promote pro- or anti- inflammatory immune environments in the CNS. There are various mechanisms underlying these effects, but it is thought that short-chain fatty acid production in the gut is of major importance (94). In turn, these anti-inflammatory products are the result of metabolism of gut contents—predominantly dietary fiber—by specific categories of bacteria, notably *Prevotellacae* and *Faecalibactirum prausnitzii*, although there are undoubtedly almost unquantifiable numbers of bacteria (and other microbes) that also play roles in regulating systemic immune responses.

More recently, evidence has accrued that in dogs with MUO (95), as well as in people with MS (96), there is relatively low abundance of these anti-inflammatory bacteria in the gut microbiome, raising the question as to whether some type of microbial transfer, or possibly even a diet change, might aid in control of both diseases. Initially, fecal microbial transfer was carried out by enema or similar methods (97) but, more recently, oral delivery of lyophilized and selected bacteria has become more widely available and is less cumbersome to administer (98). A current clinical trial (Jeffery, unpublished data) in dogs is investigating whether fecal microbial transfer might aid control of MUO in dogs (Table 2 illustrates the normalization of fecal microbiome associated with fecal microbial transfer).

**Table 2:** Example results of change in the fecal microbiome in a single dog with MUO.

	**Reference interval**	**Baseline**	**3 months**
Blautia	9.5–11.0	9.8	10.1
Cl. hiranonis	5.1–7.1	0.1	6.4
E. Coli	0.9–8.0	2.9	4.7
Faecalibacterium	3.4–8.0	2.6	4.3
Fusobacterium	7.0–10.3	10.2	9.9
Streptococcus	1.9–8.0	3.4	3.3
Turicibacter	4.6–8.1	4.5	4.6
Dysbiosis index	<0	1.8	−5.1

Bacterial abundance is tabulated at baseline and following 3 months' daily oral fecal microbial transfer from a donor dog.

Bacterial abundance data is expressed in log DNA; dysbiosis index is a ratio (https://vetmed.tamu.edu/gilab/service/assays/canine-microbiota-dysbiosis-index/).

#### Progress in diagnostic testing

Currently, diagnosis of MUO is largely presumptive and relies on cross-sectional imaging such as MRI and analysis of CSF, as reviewed elsewhere (38, 46). Definitive diagnosis requires biopsy and analysis of inflammatory brain lesions but, despite being safe (99–101), this is costly and so seldom used. Magnetic resonance imaging also remains imperfect with some studies finding that as many as 40% of lesion are not detected and there are large variations in lesion appearance [reviewed by (46)]. An interesting approach to use MRI for more precise diagnosis is to determine the permeability of the blood-brain barrier in dogs with MUO (102).

Imaging is best complemented with analysis of CSF, and a myriad of biomarkers (e.g., acute phase proteins, antibodies and cytokines) have been investigated in the last decade in an attempt to identify and quantify the molecules that might help classify and identify the different forms of MUO. However, this approach remains laborious (a few biomarkers out of thousands of possible candidates are typically tested at one time on a small number of affected dogs), access to CNS tissue to compare circulating and *in situ* biomarkers is rare, and most markers lack specificity, as highlighted by Andersen-Ranberg et al. (103). Since that review, five further studies (104–108) revisited blood neutrophil-to-lymphocyte ratio or various biomarkers that showed promise. In general, many biomarker comparisons have been made but few studies have examined sensitivity and specificity (rather than simple statistical tests of difference). Sometimes it may also be difficult to determine whether the elevation of specific biomarkers coincide or precede clinical deterioration, thereby limiting their diagnostic utility. A further limitation is that, with some exceptions (105), comparisons have often not been made between findings in MUO and other neurological conditions. On the other hand, the more sophisticated technique of “immunosignaturing,” which determines patterns of serum antibody binding to a large array of peptides, was shown to discriminate between healthy dogs, dogs with brain tumors and dogs with MUO, including in a validation set (109), suggesting promise in discriminating different types of MUO in future (see below).

#### Discrimination of types of MUO using immune profiling

**Figure 1:**
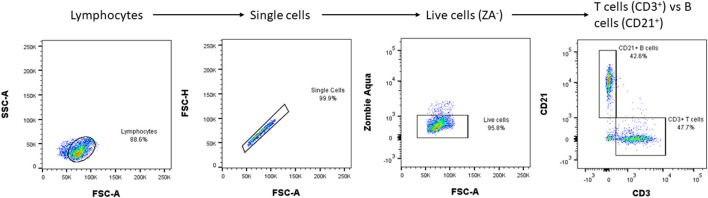
An example of CSF flow cytometry results in a dog with MUO: a population of live lymphocytes is sorted, and two antibodies used to differentiate their subtypes. The procedure requires the CSF to be centrifuged and the resultant cells to be washed and then suspended in phosphate buffer saline with fetal calf serum before being incubated with primary antibodies (e.g., CD3, CD21) that are fluorescently labeled. Subsequent flow cytometry classifies the cells according to their fluorescence, size and shape. With further validation, CSF flow cytometry may help to define the relative importance of B- vs. T- lymphocyte-mediated inflammation and so guide the choice of immunosuppressive medication [e.g., cyclosporine mainly has suppressive effect on T-lymphocytes (113)] and, in future, guide development of appropriate monoclonal antibodies against specific types of lymphocytes. SSC, side scatter; FSC, forward scatter.

In order to more precisely tailor immunotherapy for each individual dog (and to design more targeted clinical trials) it would be helpful to define the nature of the disease in each individual. For instance, to define an immune phenotype, based on the balance between B and T cell responses, or suggest more precise therapy based on the balance between T-helper and natural killer cells. Such discrimination might be made by examining the phenotypes of the cells available in the CSF. This approach, termed immune profiling, is well-known in neuro-inflammatory diseases and MS in humans (110, 111) and flow cytometry (112) is used to label and characterize inflammatory cells in the CSF. This technique has now been developed for CSF from MUO cases (Figure 1, courtesy of Prof. Wooldrige and Dr. Milodowski) and is currently being investigated to determine whether specific patterns of response are associated with specific disease types or prognosis. In time, this may allow more complex analysis of T-cell receptor usage and antigen specificity along with the study of genes leading to expression of specific receptors. This “genotyping” of the immune response together with screening tools derived from the “omics” [e.g., genomics, proteomics, metabolomics (114)] may help identify disease triggers and therefore target therapy. A concrete example is the use of plasmapheresis in humans to treat encephalitis with known antibodies directed against intracellular onconeuronal antigens such as ANNA-1/anti-Hu, and representing markers of T-cell mediated immunity (115).

#### Clinical trials to compare therapies

Since our previous review on MUO in dogs there has still only been one prospective clinical trial [on “COP” vs. cytarabine (116)] to compare therapies for MUO and that investigated only a small population of affected dogs (although it did strongly suggest one of the options was not useful). There does appear to be a widespread resistance to trials on this condition, which perhaps partly reflects the overall reluctance of veterinarians to engage with randomization but might also be because each dog can be treated as an “n-of-1” therapeutic trial (a single subject clinical trial in which an individual patient is the sole unit of observation in a study investigating the effect of a treatment). Nevertheless, it would still be helpful to know which was the best therapy to start with, or to know whether it is preferable to commence therapy with two medications rather than just one.

##### Trial outcome measures

A basic requirement of a clinical trial is to have a reliable outcome measure. For MUO a common reported end-point has been survival. This is indeed an important outcome but death from MUO may happen many months, if not years, following diagnosis, which can make it difficult to avoid large losses to follow-up. An alternative is to record neurologic deficits, but that then raises the question of how to compare heterogeneous patients with widely varying presenting signs. A solution that we have suggested previously (116) is a simple scoring scheme that aims to capture clinically-important worsening of disease and then using that outcome to declare a dog “worse” than when it presented and therefore a “failure of treatment.” The purpose of this alternative measure is that the “failure” of a treatment will occur long before the final demise of the animal and so will speed attainment of meaningful end-points to compare effectiveness between therapies. Gonçalves et al. (117) proposed recently a neurodisability scale for dogs with MUO, designed from the retrospective study of 100 cases and then tested prospectively on 31 new cases, with apparent good reliability. An alternative approach, that has been exploited in a recent unpublished trial (Harcourt-Brown, personal communication) is to compare the response to different treatment regimens over a very short follow-up period. This has the twin merits of drastically reducing loss to follow-up and also identifying interventions that protect dogs from dying in the immediate period after diagnosis, when a large proportion of overall mortality occurs.

## Conclusion

During the past decade many reports on treatment of MUO have appeared but, unfortunately, it is hard to draw strong overall conclusions from these studies and no single medication appears advantageous over others, as evidenced by the enormous survival range of a few days to hundreds of days regardless of treatment protocol.

This impasse is in part due to the lack of comparison of medications in randomized controlled trials, but possibly also to the use of survival as the outcome measure, which may prevent detection of more subtle differential medication benefits. It therefore appears that examining response to treatment in the future should also rely on scoring systems, as previously proposed and currently in further development.

The lack of discernible differences between treatments may be because of a mixture of “responders” and “non-responders” in each cohort of dogs, and the design of future trials might be enhanced by identifying specific patient profiles or combination of baseline variables that could be used for stratification. Immune profiling of MUO cases (e.g., using CSF flow cytometry) would be especially powerful in this regard because it permits: (i) splitting canine MUO into more distinct groups (thus stratifying for trials); and (ii) *post-hoc* analysis of treatment outcomes in light of the immune footprint. In addition, such in-depth disease analysis may identify pathophysiological mechanisms in MUO and potential triggers. New approaches to therapy might then become more apparent, in particular those that can address disease triggers rather than treat the end-stage of the disease as we may have been doing so far.

## Author contributions

NJ: format of article, writing manuscript, and literature search and analysis of published data, Table 2. NG: writing the manuscript and literature search and analysis of published literature, Table 1 and Figure 1. NJ and NG contributed to and approved the final manuscript, figure, and tables.
